# Direct Ink Writing 3D Printing of Anisotropic Superhydrophobic/Superelastic Porous Silicone Rubber for Recyclable Dichloromethane Absorption

**DOI:** 10.3390/polym18212714

**Published:** 2026-11-02

**Authors:** Longtao Ge, Zhoukun He, Lin Deng, Zhijun Wang, Qiang Guo, David Hui, Wei Feng, Qingyuan Wang, Xiaorong Lan, Jiayan Mao

**Affiliations:** 1 School of Mechanical Engineering, Chengdu University, Chengdu 610106, China; gelongtao2026@163.com (L.G.); hezhoukun@cdu.edu.cn (Z.H.); 17743351225@163.com (L.D.); fengwei@cdu.edu.cn (W.F.); 2 Institute for Advanced Study, Research Center of Composites & Surface and Interface Engineering, Chengdu University, Chengdu 610106, China; wangzhijun@cdu.edu.cn; 3 Luzhou Key Laboratory of Oral & Maxillofacial Reconstruction and Regeneration, The Affiliated Stomatological Hospital, Southwest Medical University, Luzhou 646000, China; 15282609735@163.com; 4 Institute of Stomatology, Southwest Medical University, Luzhou 646000, China; 5 Department of Mechanical Engineering, LSU New Orleans, New Orleans, LA 70148, USA; dhui@uno.edu; 6 Failure Mechanics and Engineering Disaster Prevention Key Laboratory of Sichuan Province, Sichuan University, Chengdu 610065, China; 7 Basic Medicine Research Innovation Center for Cardiometabolic Diseases, Ministry of Education, Southwest Medical University, Luzhou 646000, China; 8 Metabolic Vascular Diseases Key Laboratory of Sichuan Province, Southwest Medical University, Luzhou 646000, China; 9 Department of Nephrology and Institute of Nephrology, Sichuan Provincial People’s Hospital, School of Medicine, University of Electronic Science and Technology of China, Sichuan Clinical Research Centre for Kidney Diseases, Chengdu 610072, China

**Keywords:** anisotropic surface wettability, superhydrophobic, DCM absorption, 3D printing, porous structure, polydimethylsiloxane, silicone rubber

## Abstract

Special wettability on biological surfaces generally arises from the synergy between hierarchical microstructures and surface chemistry, providing inspiration for biomimetic superhydrophobic materials. However, conventional fabrication strategies often involve complex procedures or expensive molds, limiting the precise construction of ordered microstructures. Herein, direct ink writing (DIW) 3D printing was used to fabricate polydimethylsiloxane (PDMS)-based porous silicone rubber with integrated anisotropic surface wettability (ASW), superhydrophobicity, superelasticity, and dichloromethane (DCM) absorption performance. By regulating printing speed, pneumatic pressure, and nozzle angle, porous silicone rubber with well-defined anisotropic microstructures was constructed. The DIW-printed porous silicone rubber exhibited pronounced ASW and superhydrophobicity. The material maintained structural integrity under compressive strain and rapidly recovered its original shape after unloading. After 1000 cycles of tensile and compressive loading, the tensile strength decreased while the compressive strength showed no significant degradation, demonstrating excellent superelasticity and low energy dissipation. The DIW sample showed faster DCM uptake within 30 min, compared with conventional hot-pressed PDMS materials, whereas the equilibrium adsorption capacities of both materials after one day of exposure were comparable. Meanwhile, the DIW sample exhibited excellent stability during cyclic DCM absorption. In addition, the material maintained stable DCM absorption performance under contaminated conditions and at different temperatures. This study provides a new strategy for fabricating multifunctional intelligent porous elastomers via 3D printing, and the prepared material shows possible applications in DCM–water separation and potential applications in waterproof materials and porous biomedical materials.

## 1. Introduction

In nature, special surface wettability, such as superhydrophobicity and anisotropic surface wettability (ASW), is widely observed on biological surfaces, including lotus leaves [1], rice leaves [2] and butterfly wings [3], endowing them with unique functions such as self-cleaning, directional liquid transport, and anti-adhesion. Inspired by these natural surfaces, elastomeric materials with artificially constructed special wettability have shown great application potential in organic pollutant–water separation, waterproof and breathable materials, flexible sensors, and biomedical materials [4,5,6,7,8,9,10,11,12,13,14,15,16,17,18,19,20,21,22]. Currently, materials used for adsorbing organic pollutants are mainly categorized into organosilicon compounds, polymers, organic monomers, carbon-based materials, and aerogels [23,24,25,26,27,28]. Among them, silicone rubber, especially polydimethylsiloxane (PDMS), has become an ideal matrix for fabricating such functional materials because of its intrinsic low surface energy, excellent chemical stability, biocompatibility, and high elasticity [8,29]. However, conventional fabrication of silicone rubber usually relies on processes such as compression molding, chemical foaming, and template-assisted methods. These methods often require complex mold design and tedious multistep post-treatment, while making it difficult to precisely regulate micro-/macroscopic porous structures [30,31,32,33], thereby limiting the synergistic optimization of multiple properties, including wettability and mechanical performance.

In recent years, additive manufacturing (AM) [34,35], especially direct ink writing (DIW), has provided a new strategy for fabricating porous silicone rubber with precisely tunable structure–property relationships because of its mold-free processing, programmable design, and capability to rapidly construct complex three-dimensional architectures [36,37,38,39,40]. Through DIW, the surface texture and internal pore architecture of materials can be precisely designed at submillimeter or even micrometer scales, thereby enabling the regulation of multiple properties, including wettability, mechanical performance, and organic pollutant absorption capability. For example, Xiao et al. regulated the elastic modulus from 0.06 MPa to more than 0.8 MPa by adjusting the filament orientation between adjacent layers from 30° to 90° and the filament spacing during printing from 0.8 mm to 2.4 mm [41]. He et al. systematically investigated the effects of printing speed, filament diameter (FD), and filament spacing (FS) on surface wettability by DIW 3D printing of PDMS inks, and fabricated PDMS films with a water contact angle (WCA) of approximately 155°, which exhibited superhydrophobicity and ASW behavior [42]. Chen et al. combined DIW with a salt-template pore-forming strategy to prepare silicone rubber foams with a trimodal porous structure [36]. The resulting material exhibited excellent superelasticity, withstanding 90% compressive strain without obvious plastic deformation, as well as superhydrophobicity with a WCA above 150° and superior trichloromethane (CHCl3) absorption capability. Beyond organic pollutant absorption and wettability regulation, the precise structural programmability enabled by DIW also suggests potential value in other porous membrane systems [43,44,45,46]. After appropriate surface modification, such controllable porous architectures may provide a promising structural basis for the future development of porous membranes for hemodialysis applications [44,45,47]. Compared with currently used hemodialysis membranes based on polysulfone (PSf) or polyethersulfone (PES) [48,49,50], whose pore structures are mainly governed by phase-separation processes, DIW-based fabrication offers greater flexibility in regulating pore geometry, pore connectivity, and anisotropic transport pathways. This advantage may facilitate the rational design of next-generation multifunctional porous membranes in possible future directions, such as hemodialysis membranes and artificial heart valves, with improved mass transfer efficiency, mechanical robustness, and interface-related performance. Although there are some typical PDMS-based porous materials for dichloromethane (DCM)–water separation reported in recent years (Table S1 in the Supporting Information) [51,52,53,54], and these studies have made important progress in the geometric regulation of wettability and the development of superelastic fouling–absorbing materials, few studies have reported the integration of ASW, superelasticity, and recyclable fouling absorption into a single 3D-printed porous silicone rubber system.

This study aims to reveal the relationship between printing parameters and geometric structures, fabricate ordered porous silicone rubber, and further develop a multifunctional porous silicone rubber with special wettability, superelasticity, and recyclable DCM absorption (Figure S1A in the Supporting Information).

## 2. Materials and Methods

### 2.1. Materials

PDMS (SE 1700, viscosity: 542 Pa·s) and its corresponding curing agent were purchased from Dow Corning Corporation, Midland, MI, USA, and used at a weight ratio of 10:1. 1H,1H,2H,2H-perfluorooctyltrichlorosilane (CF_3_(CF_2_)_5_CH_2_CH_2_SiCl_3_, PFTS, 97%) and 3-butyn-1-ol (97%) were purchased from Shanghai Haohong Scientific Co., Ltd. (Shanghai, China) and Shanghai Aladdin Bio-Chem Technology Co., Ltd. (Shanghai, China), respectively. Dichloromethane (DCM, MW: 84.93, analytical grade) was obtained from Chengdu Chron Chemical Co., Ltd. (Chengdu, China) Sudan IV (C_24_H_20_N_4_O, MW: 380.44) was purchased from Shanghai Macklin Biochemical Co., Ltd. (Shanghai, China) Sodium dodecyl sulfate (SDS, C_12_H_25_NaO_4_S) was obtained from Shanghai Haohong Biomedical Technology Co., Ltd. (Shanghai, China).

### 2.2. Preparation of PDMS Ink

First, 20 g of SE 1700, 2 g of curing agent, and 0.2 g of 3-butyn-1-ol were mixed under room conditions using a gravity mixer at a speed of 700 rpm for 30 min. The resulting mixture was then transferred into a syringe barrel. Finally, the syringe barrel was sealed with an end cap and centrifuged at 8000 rpm for 20 min to further remove trapped air bubbles.

### 2.3. DIW 3D Printing and Thermal Curing

The syringe loaded with the prepared ink was mounted on a custom-built 3D printer. The 3D printer consisted of a computer-controlled three-axis motion platform, a fluid dispenser equipped with a screw extrusion system, and a micronozzle. The micronozzle was programmed to move along the X-, Y-, and Z-axes while extruding the PDMS ink according to the preset printing parameters.

To facilitate the peeling of the cured PDMS samples from the substrate, the glass substrate was hydrophobically treated with a 1% PFTS solution for 1 min before PDMS ink printing, followed by drying at 80 °C for 5 h.

The FS was set to 0.8 mm. The micronozzle with an inner diameter of 260 μm was lifted by 0.4 mm along the *Z*-axis to continue printing polymer filaments in the *Y*-axis direction, thereby forming the next layer. In this way, continuous and well-aligned PDMS polymer filament structures were deposited on the glass substrate under different printing parameters, including printing speed and pneumatic pressure. Finally, an ordered anisotropic grid pattern with a four-layer height and grooved structure was obtained. The substrate carrying the 3D-printed porous silicone rubber was then heated at 120 °C for 1 h. The PDMS control sample was prepared by hot pressing at 120 °C for 1 h after the prepared ink was placed in a 50 × 50 × 2 mm hot-pressing mold (Figure S1B).

After thermal curing, the printed sample and the control sample were peeled from the substrate for subsequent characterization and performance evaluation.

### 2.4. Characterization

#### 2.4.1. Surface Wettability

The WCA of the sample surface was measured at ambient temperature using an SDC-35 contact angle goniometer (Dongguan Shengding Precision Instrument Co., Ltd., Guangdong, China). A 5 μL deionized water droplet was placed on the sample surface, and the WCA values were measured along the Y direction (θ_∥_) and X direction (θ_⊥_). Here, θ_⊥_ and θ_∥_ represent the WCA measured perpendicular and parallel to the PDMS filaments, respectively. The absolute WCA difference between the perpendicular and parallel directions, defined as Δθ = |θ_⊥_ − θ_∥_|, was used to evaluate the degree of wetting anisotropy of the porous PDMS films.

#### 2.4.2. Physical Structure

The surface morphology and structural parameters of the porous silicone rubber were observed using a BMC500 optical digital microscope (Phenix Optics Co., Ltd., Shangrao, China) and a ZEISS Sigma 300 scanning electron microscope (SEM, ZEISS, Oberkochen, Germany).

#### 2.4.3. Mechanical Properties

The mechanical properties of PDMS were tested using a JHY-5000 microcomputer-controlled universal testing machine (Xiamen Jinheyuan Technology Co., Ltd., Xiamen, China). For the tensile test, PDMS samples with a width of 10 mm and a length of 70 mm were placed between the grips, fixed, and stretched at a rate of 500 mm/min until fracture. For the cyclic tensile test, a 30 mm section at the center of the sample was marked with a marker pen. Then, it was slowly stretched until 100% was reached and then allowed to recover to its original state, which was defined as one cycle. After 1000 cycles, the tensile test was conducted once again using the same testing machine.

For the compression test, cubic PDMS samples with a side length of 10 mm were placed between self-aligning compression platens and compressed at a rate of 10 mm/min until reaching the maximum compression limit. For the cyclic compression test, a cube with a side length of 10 mm was slowly compressed to 30% of its original height and then allowed to recover to its original state, which was defined as one cycle. After 1000 cycles, the compression test was conducted once again using the same testing machine.

#### 2.4.4. DCM Absorption Capacity

Different samples were immersed in 10 mL of DCM, and their DCM absorption capacity was measured at different immersion times. The DCM absorption capacity was calculated according to the following equation:

$$\mathrm{DCM}\;\mathrm{absorption}\;\mathrm{capacity}\;(\%)\;=\;\frac{m_{t}-m_{0}}{m_{0}}\times 100\%$$

where *m_t_* and *m*_0_ represent the mass of the sample after and before DCM absorption, respectively.

For staining of DCM, 0.05 g of Sudan IV was mixed with 10 mL of DCM and stirred at 500 rpm for 30 min using a magnetic stirrer.

DCM emulsion was prepared by mixing 1 mL of DCM, 10 mL of ultrapure (UP) water, and 500 mg of SDS, followed by magnetic stirring at 500 rpm for 30 min.

#### 2.4.5. Effect of Environmental Conditions on DCM Absorption Performance

Pollutant environment treatment:

To simulate the influence of a complex pollutant environment on the DCM absorption performance of the material during practical DCM–water separation, a multicomponent pollutant system was constructed. Specifically, 40 g of DCM, 5 g of pepper powder, 10 g of potato starch, 1 g of chalk dust, 5 g of laundry detergent, 2 g of hand soap, 10 g of ink, 20 g of deionized water, and 5 g of NaCl were added to a glass container and thoroughly mixed to form a simulated pollutant environment. Subsequently, porous PDMS samples with dimensions of 10 mm × 10 mm were immersed in the above pollutant system and treated at room temperature for 12 h. After treatment, the samples were removed, cleaned, and then tested for their DCM absorption performance to evaluate the effect of the pollutant environment on the DCM absorption capacity of the material.

Temperature treatment:

To investigate the influence of temperature variation on the DCM absorption performance of porous PDMS, samples with dimensions of 10 mm × 10 mm were placed in an oven at 80 °C and a refrigerator at −20 °C for 12 h, respectively. After high-temperature and low-temperature treatments, the samples were allowed to recover to room temperature. Their DCM absorption capacity was then measured using the same method and compared with that of untreated samples.

Absorption performance of SDS-emulsified DCM in water:

The absorption microscopic images of SDS-emulsified DCM in water were observed using a BMC500 optical digital microscope (Phenix Optics Co., Ltd., Shangrao, China).

#### 2.4.6. Statistics and Analysis

For statistical analysis of water contact angle, DCM absorption, and mechanical properties, each sample was tested three times, and the obtained data were expressed as the mean ± standard deviation.

## 3. Results and Discussion

### 3.1. Optimization of the 3D Printing Process

#### 3.1.1. Linear Relationship Between Printing Speed and FD

During DIW 3D printing, the PDMS ink exhibited good printability and could be extruded into continuous filaments, thereby enabling the assembly of desirable porous structures without collapse. Printing speed is a critical processing parameter in DIW, as it significantly affects the forming quality and structural accuracy of PDMS materials. An excessively high printing speed may lead to severe interlayer deformation and structural distortion, whereas an overly low printing speed may result in ink accumulation and over-extrusion, thereby reducing dimensional fidelity [41,55]. Because differences in component precision and program control exist among different printers, it was necessary to first systematically investigate the filament morphology and FD under different printing speeds in this study, so as to provide an accurate reference for subsequent structural design and printing fabrication using the present printer.

**Figure 1:**
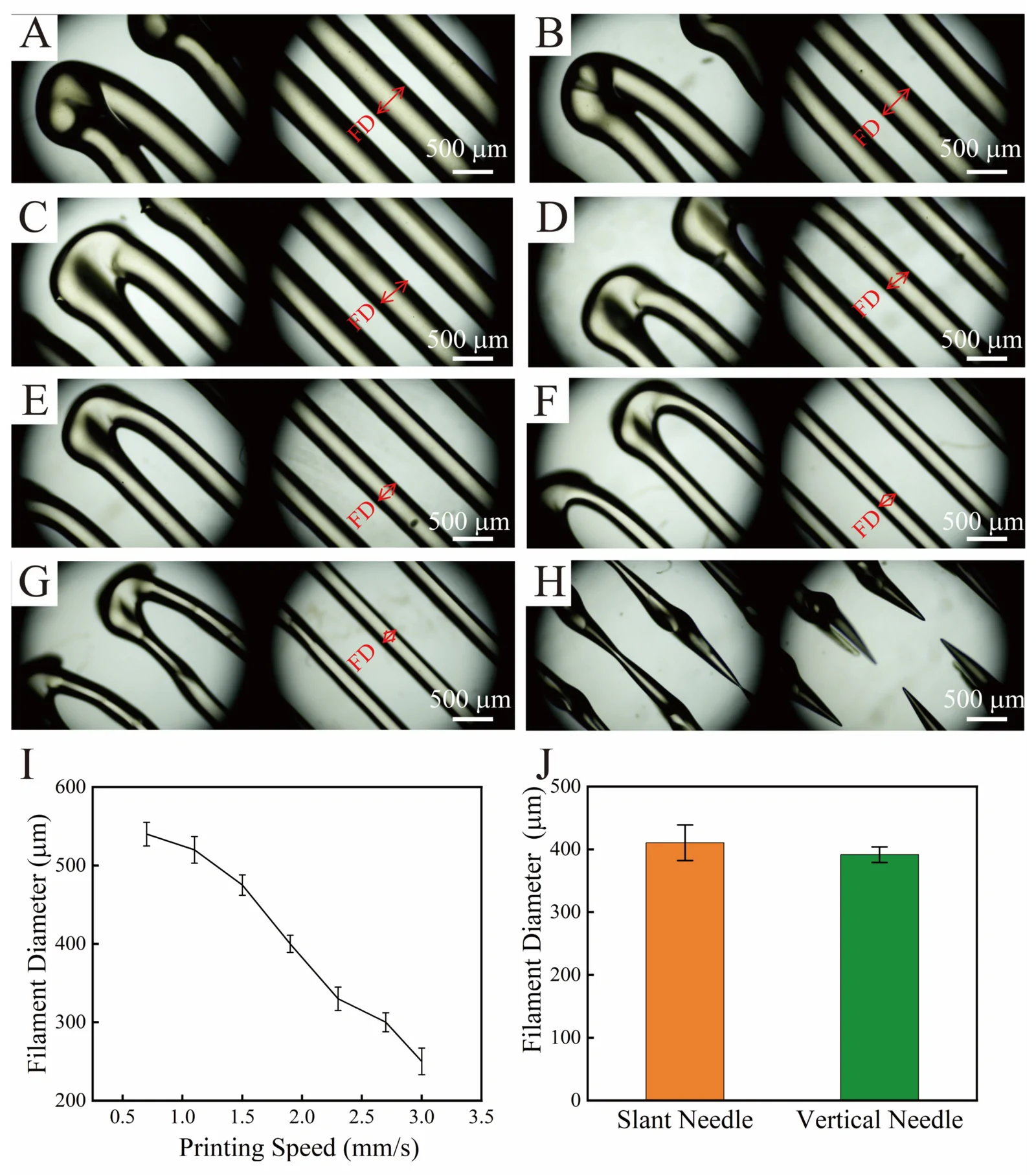
The morphology and FD (showed as red double-headed arrow) of filaments at different printing speeds (**A**–**H**); filament diameter as a function of printing speed (**I**); comparison of FD between the slant nozzle and vertical nozzle (**J**).

Figure 1 shows the FD of PDMS printed at different printing speeds under a pneumatic pressure of 2.5 bar. As observed in Figure 1A,B, at relatively low printing speeds of 0.7 and 1.1 mm/s, excessive material accumulation occurred at the corner regions. The individual printed lines were obviously wider and less uniform. Moreover, because of the excessive material deposition per unit length, the spacing between adjacent filaments was partially filled, resulting in smaller effective pores. As shown in Figure 1C–F, when the printing speed ranged from 1.5 to 2.7 mm/s, the corner regions became more regular, suggesting that the printing speed and ink extrusion rate gradually reached a balanced state near 1.5 mm/s. Meanwhile, the FD gradually decreased as the printing speed increased. In Figure 1G, at a printing speed of 3.0 mm/s, filament deformation was observed at the corner region, indicating that this speed was close to the critical point of filament breakage. In Figure 1H, filament breakage was clearly observed at a printing speed of 4.0 mm/s. As shown in Figure 1I, the FD gradually decreased with increasing printing speed until filament breakage occurred, indicating an inverse relationship between printing speed and FD.

Compared with our previously reported relationship between FD and printing speed for samples fabricated using a different printer [42], the present results further indicate that although the exact quantitative correspondence between FD and printing speed may vary because of inherent differences in component precision and program control among different printers, the overall trend that FD decreases with increasing printing speed remains consistent. This further confirms the stability and reproducibility of our study. According to previous results, when the FD was approximately 400 μm, the printed structure exhibited favorable hydrophobic performance and achieved superhydrophobicity. Therefore, based on the results shown in Figure 1I, a printing speed of 1.9 mm/s was selected for sample fabrication in the following experiments.

#### 3.1.2. Relationship Between Pneumatic Pressure and Pore Morphology

In DIW printing, in addition to printing speed, pneumatic pressure is another key processing parameter that affects printing quality and pore morphology. Pneumatic pressure is positively correlated with the ink extrusion rate and consequently influences the volume of ink extruded per unit time [55]. An excessively low pneumatic pressure reduces printing efficiency. Therefore, in this study, pneumatic pressures ranging from 2.5 to 4.0 bar were selected. A nozzle with an inner diameter of 260 μm was used, and the ink was deposited layer by layer along the preset X- and *Y*-axis paths, with the *Z*-axis lifted by 0.40 mm for each layer. Four-layer ordered grid structures were printed at a printing speed of 1.9 mm/s, thereby obtaining a series of porous PDMS samples fabricated under different pneumatic pressures.

The structures of porous silicone rubber obtained under different pneumatic pressures are shown in Figure S2A–F (scale bar: 500 μm), where the red boxed regions indicate the non-overlapping areas between adjacent layers. When the pneumatic pressure was 4.0 bar (Figure S2A), the non-overlapping area was relatively large, and the interlayer overlap was low. This may be attributed to the excessively high extrusion rate caused by the high pneumatic pressure, which led to over-deposition and deformation of the filaments. At pneumatic pressures of 3.7 bar (Figure S2B) and 3.4 bar (Figure S2C), the non-overlapping area did not decrease obviously, indicating that the interlayer overlap was not significantly improved. When the pneumatic pressure decreased to 3.1 bar (Figure S2D) and 2.8 bar (Figure S2E), the non-overlapping area was markedly reduced compared with those obtained at 3.4, 3.7, and 4.0 bar, suggesting that the interlayer overlap began to improve. This change can be attributed to the reduced extrusion rate caused by the decreased pneumatic pressure, which gradually matched the printing speed. When the pneumatic pressure was further reduced to 2.5 bar (Figure S2F), the non-overlapping area became the smallest and the best interlayer overlap was achieved, indicating that the extrusion rate at this pneumatic pressure was most compatible with the printing speed of 1.9 mm/s. Therefore, a pneumatic pressure of 2.5 bar was used for subsequent sample fabrication.

Overall, pneumatic pressure plays an important role in regulating the overall performance of porous silicone rubber by controlling the extrusion rate and subsequently affecting FD and pore morphology. Within a certain range, reducing pneumatic pressure is beneficial for decreasing FD and increasing porosity, thereby improving the pore structure of porous silicone rubber.

#### 3.1.3. Relationship Between Nozzle Angle and Pore Morphology

In addition, under the experimental conditions of a printing speed of 1.9 mm/s and a pneumatic pressure of 2.5 bar, the structural differences between samples printed using a vertical nozzle, where the nozzle was perpendicular to the printing platform at 90°, and a slant nozzle, where the nozzle formed an acute angle of 60° with the printing platform, were further investigated. For the samples printed using the slant nozzle, the FD ranged from 382 to 439 μm, with a range of 57 μm. In contrast, for the samples printed using the vertical nozzle, the FD ranged from 379 to 404 μm, with a smaller range of 25 μm, indicating that the vertical nozzle method could effectively reduce the errors between the filament diameters (Figure 1J). Moreover, when the slant nozzle was used, the non-overlapping area between adjacent layers, as indicated by the red boxed region in Figure S2G, was relatively large. By contrast, when the nozzle was perpendicular to the printing platform, the FD variation among different filaments was smaller, and the non-overlapping area between adjacent layers, as indicated by the red boxed region in Figure S2H, was also reduced. The less uniform filaments printed using the slant nozzle may be mainly attributed to the uneven distribution of extrusion pressure in different directions and the nonuniform contact angle between the extruded filament and the substrate. Therefore, the vertical nozzle was used for sample fabrication in the following experiments.

### 3.2. SEM Analysis of DIW-Printed Porous PDMS

Based on the preceding optimization of processing parameters, the optimal printing conditions were determined as follows: printing speed of 1.9 mm/s, pneumatic pressure of 2.5 bar, nozzle perpendicular to the printing plane, *Z*-axis lifting height of 0.40 mm per layer, and nozzle inner diameter of 260 μm. Under these printing conditions, four-layer porous PDMS samples were fabricated for the subsequent performance evaluation.

**Figure 2:**
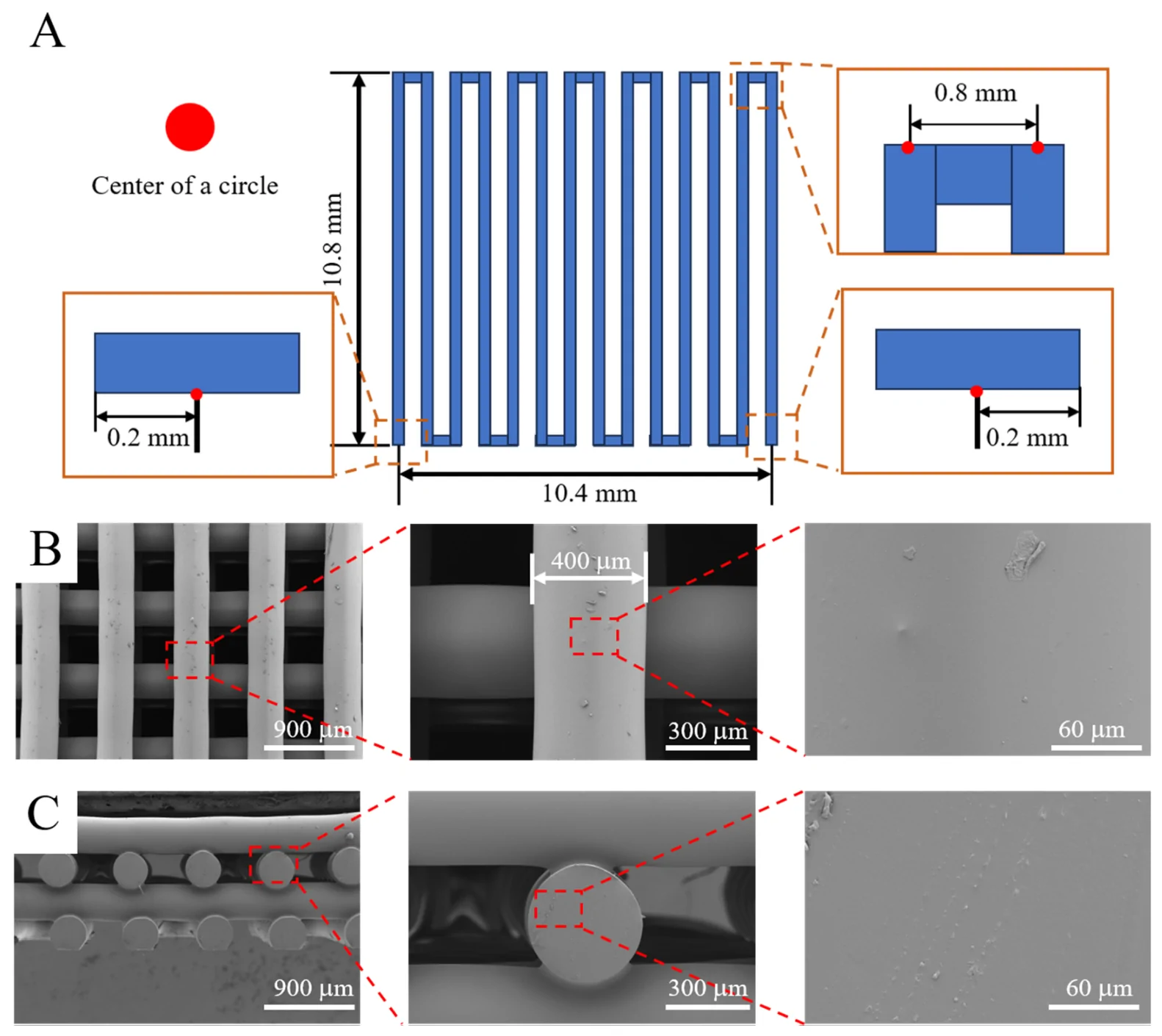
Schematic representation (**A**) and SEM images of DIW-printed porous PDMS (**B**,**C**) ((**B**): surface morphology, (**C**): cross-sectional morphology).

Under the optimized printing parameters, the filaments were deposited under the guidance of a computer-controlled program, with each layer oriented perpendicular to the underlying filaments, thereby forming a typical anisotropic physical architecture. Since the printing paths between adjacent layers only underwent a 90° rotation without changing the geometric parameters of the single-layer structure, each printing layer had the same porosity. Therefore, only the porosity of the single-layer structure needed to be calculated to obtain the porosity of the overall three-dimensional structure. Taking the structure shown in Figure 2A as an example, the distance between the centers of the leftmost and rightmost cylindrical filaments in the single-layer printing path was 10.4 mm, the filament center spacing was 0.8 mm, and there were a total of 14 parallelly arranged filaments. In addition, the adjacent filaments were connected through connection areas, and each connection area had a length of 0.8 mm, with a total of 13 connection areas. Therefore, the total length of the PDMS solid part in this single-layer structure could be expressed as follows: the total filament length was calculated from 14 filaments and 13 connecting segments, giving a filament volume of (13 * 0.8 + 14 * 10.4) * π * 0.2^2^ ≈ 19.6 mm^3^. The total volume of this layer was approximated as a rectangular solid with a length, width, and height of 10.8 *, 10.8 *, and 0.4 ≈ 46.7 mm^3^. Therefore, the porosity of the porous PDMS printed by DIW was estimated to be ((46.7–19.6)/46.7) * 100% ≈ 58%. As observed from the surface morphology (Figure 2B), highly ordered microchannel structures induced by the printing path were formed, and the surface was relatively smooth. The cross-sectional morphology (Figure 2C) revealed an interconnected porous network and periodically arranged structural units, with a relatively smooth cross section. In addition, the good interfacial bonding between filaments in adjacent layers ensured the mechanical stability of the printed samples, providing a solid structural basis for subsequent stability tests. Furthermore, the SEM images of the surface and cross section of porous PDMS showed numerous submillimeter grooves, which are considered an effective structural feature for achieving ASW. This anisotropic porous architecture can provide directional liquid transport channels and further regulate the anisotropic wetting behavior of the PDMS surface.

### 3.3. WCA Analysis of Porous Silicone Rubber Materials

After confirming the DIW printing parameters, the WCA values of PDMS samples prepared by the two different methods were measured to evaluate their surface wettability. As shown in Figure S3A, the WCA values of DIW-printed PDMS were measured in the X and Y directions, respectively. The X direction exhibited pronounced hydrophobicity, with an initial WCA as high as 151.1 ± 1.2°, reaching the superhydrophobic state. After 10 min, the WCA gradually decreased to 142.2 ± 2.2° because of water droplet evaporation, corresponding to a decrease of 8.9°. The sustained high WCA above 140° indicated that the surface maintained a relatively stable hydrophobic state. In contrast, the initial WCA in the Y direction was significantly lower, reaching 125.2 ± 0.7°, which corresponded to hydrophobicity rather than superhydrophobicity. During the 10 min observation period, the WCA in the Y direction showed a continuous decreasing trend, with a decrease of 9.5°. The significant difference in WCA between the X and Y directions demonstrated that the strong ASW of the DIW-printed PDMS surface resulted from the anisotropic structures of surface (Figure 2B), which represents one of the advantages of the DIW printing strategy.

**Figure 3:**
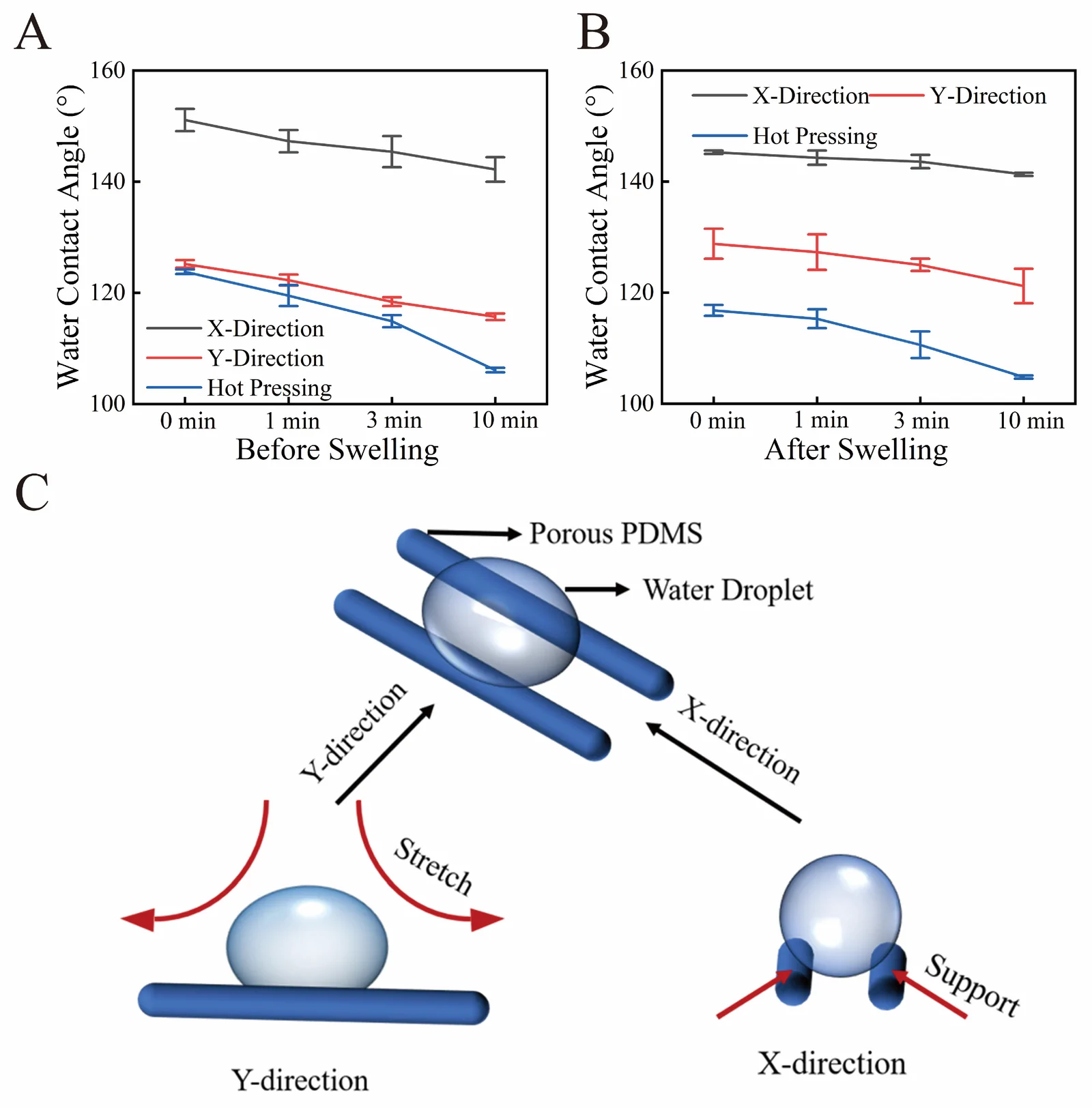
Time-dependent WCA changes for different samples before (**A**) and after (**B**) swelling in DCM. Schematic diagram of the wetting mechanism of DIW-printed porous PDMS (**C**).

The WCA of PDMS prepared by hot pressing was similar to that measured in the Y direction of the DIW-printed sample, with an initial value of 123.8 ± 0.4° (Figure S3A). This can be attributed to the absence of printed filament structures on the hot-pressed PDMS surface, which otherwise contribute to increased surface roughness in DIW-printed PDMS. Therefore, water droplets tended to spread more readily on the relatively smooth surface of hot-pressed PDMS under the effect of gravity, resulting in a lower WCA. In addition, the WCA of the hot-pressed PDMS decreased more obviously with time, from 123.8° to 106.1°, with a ΔWCA of approximately 17.7°. This decrease was mainly due to the lower initial WCA, which led to a larger contact area and stronger adhesion between the water droplet and the PDMS surface. Consequently, the decrease in WCA became more pronounced after water evaporation (Figure 3A).

To investigate the changes in surface wettability after DCM absorption, the two types of PDMS samples were swollen in DCM for 30 min, followed by WCA measurements (Figure S3B). For the DIW-printed PDMS, the X direction still maintained a high initial WCA of 145.3 ± 0.3°, which only slightly decreased to 141.3 ± 0.3° after 10 min, corresponding to a decrease of 4.0°. In the Y direction, the initial WCA after swelling was 128.8 ± 2.7° and decreased to 121.2 ± 3.1° after 10 min, with a decrease of 7.6°. For the hot-pressed PDMS, the initial WCA after swelling was the lowest, at 116.8 ± 1.0°, and continuously decreased to 104.8 ± 0.3°. These results indicate that, compared with the unswollen PDMS samples, the decreasing trend of WCA for both DIW-printed and hot-pressed PDMS after DCM swelling was slightly slowed down. However, for the hot-pressed PDMS, the WCA still decreased faster than those measured in both the X and Y directions of the DIW-printed PDMS. Furthermore, the results show that DCM swelling did not cause significant changes in the wettability of PDMS prepared by either method (Figure 3B).

The above results indicate that the DIW-printed porous PDMS exhibited ASW, which can be partly attributed to the parallel filaments in the top layer. These parallel filaments led to distinct differences in droplet spreading and sliding behaviors along different directions. As shown in Figure 3C, in the X direction, the supporting effect of the filaments and the suspended structural features significantly increased the resistance of droplets to spread across the filaments during WCA measurement, resulting in a higher WCA. In contrast, during WCA measurement in the Y direction, the spreading resistance of droplets along the filament grooves was markedly lower than that in the X direction, leading to a lower WCA. Consequently, the sample surface exhibited typical ASW. The structural effect illustrated in Figure 3C was further verified by rolling angle (RA) measurements. As shown in Figure S4A, when the observation direction was consistent with the X direction used for WCA measurement, namely when the DIW-printed porous PDMS was tilted along the Y direction, the water droplet had to roll across the filaments. Therefore, even when the sample was tilted to 80°, the water droplet remained stably attached to the surface without obvious rolling behavior, indicating an RA greater than 80° in the X direction. In contrast, when the observation direction was consistent with the Y direction used for WCA measurement (Figure S4B), namely when the DIW-printed porous PDMS was tilted along the X direction, obvious droplet rolling occurred at a tilt angle of 40–60°. This result was closely related to the easier droplet spreading along the filament grooves described above. Therefore, the RA in the Y direction was markedly smaller than that in the X direction, further confirming the typical ASW of the surface. These results indicate that the surface exhibited an intermediate superhydrophobic state with a certain droplet-rolling ability but not exhibiting lotus-leaf-like superhydrophobicity with an RA below 10° [56,57]. Meanwhile, it also showed an adhesive superhydrophobic feature similar to the rose-petal effect [56,57].

In the WCA measurements of DIW-printed porous PDMS, the material not only exhibited superhydrophobicity in the X direction but also showed distinct ASW. Furthermore, the DIW-printed porous PDMS material showed typical hydrophobicity (Figure S4C) and oleophilicity (Figure S4D), respectively. These characteristics indicate that DIW-printed PDMS has promising application potential in the field of DCM–water separation.

### 3.4. Mechanical Properties of Porous Silicone Rubber

**Figure 4:**
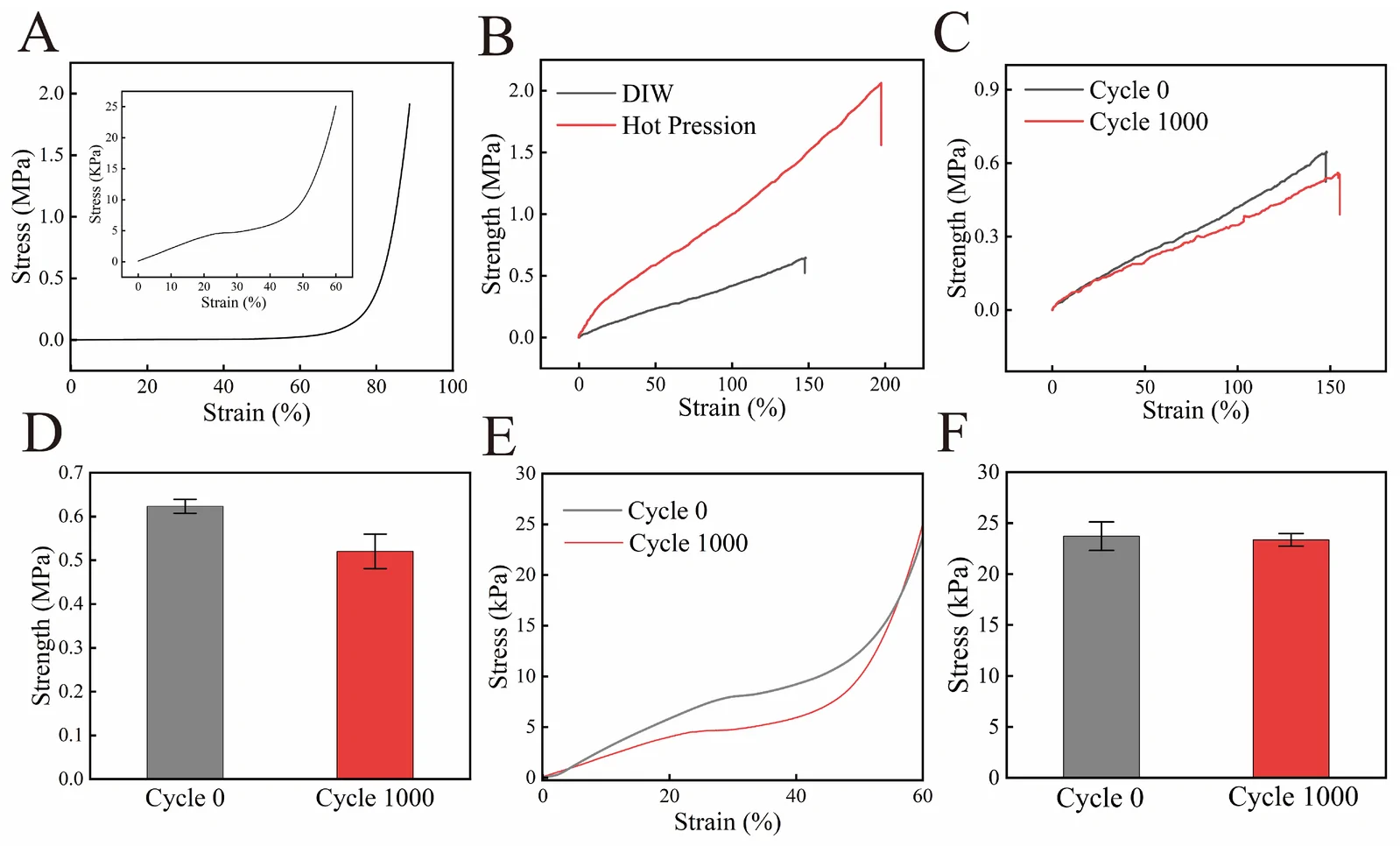
Compressive stress–strain curve of DIW-printed porous PDMS (**A**), with an inset showing the enlarged region. Tensile stress–strain curves of DIW-printed porous PDMS and hot-pressed PDMS (**B**). Tensile stress–strain curves before and after 1000 tensile loading cycles (**C**). Comparison of tensile strength (**D**). Compressive stress–strain curves before and after 1000 compressive loading cycles (**E**). Comparison of compressive strength (**F**).

In DCM–water separation applications, the mechanical properties of PDMS are also critical because they are closely related to its service life. Therefore, the mechanical performance of the samples prepared was further evaluated. As shown in Figure 4A, the compressive stress–strain curve showed that the DIW-printed porous PDMS did not exhibit a sharp stress drop or structural failure point under a compressive strain of up to 89%, presenting a typical three-stage deformation behavior, including the linear elastic region, stress plateau region, and densification region. Figure S5A shows photographs of the sample during the compression test. After unloading (Figure S5B), the sample could rapidly recover to its original size, with no obvious structural damage or residual deformation compared with the uncompressed sample, indicating excellent superelasticity and reversible deformability.

Figure 4B shows the tensile stress–strain curves of DIW-printed PDMS and hot-pressed PDMS, and Figure S5C shows photographs of the sample during the tensile test. The DIW-printed PDMS exhibited an elongation at break of 148% and a tensile strength of 0.65 MPa, whereas the hot-pressed PDMS showed an elongation at break of 197% and a tensile strength of 2.06 MPa. Clearly, both the tensile strength and elongation at break of DIW-printed PDMS were lower than those of conventional hot-pressed PDMS. This is because the ultimate tensile strength and elongation at break of porous materials are generally considered to be negatively affected by porosity [58,59,60]. The increased porosity reduced the interactions between adjacent DIW-printed PDMS filaments. Meanwhile, the filament-stacking printing mode further weakened the direct interactions among the filaments, thereby reducing the effective PDMS volume responsible for load bearing and shortening the crack propagation distance, ultimately leading to decreased tensile properties [36]. In addition, an increased number of pores can introduce more stress concentration sites, resulting in reduced material strength [61]. Furthermore, samples with dimensions of 10 × 70 mm were printed, folded in half, fixed with clamps, and loaded with a 150 g standard load suspended by a medical suture. As shown in Figure S5D, no obvious damage occurred in the material, indicating its good resistance to suture cutting, local indentation, and localized tensile deformation.

Furthermore, long-term deformation recovery experiments were performed to comparatively evaluate the shape recovery and creep resistance of samples prepared by DIW and hot pressing (Figure S5E,F). After the two types of samples were folded along their symmetry axes and continuously compressed with a 500 g load for one week, the load was removed. The DIW-printed porous PDMS sample rapidly and completely recovered to its original shape, with a flat surface and no visible residual strain. In contrast, the hot-pressed sample exhibited significant irreversible plastic deformation, with macroscopic surface indentation, and could not fully conform to the substrate. These results indicate that although the porous structure of DIW-printed PDMS reduced its tensile strength and elongation at break compared with conventional hot-pressed PDMS, it endowed the material with significantly superior elastic recovery and resistance to permanent deformation. These properties are beneficial for its repeated use in practical applications. Furthermore, to evaluate the structural stability and reusability of DIW-printed porous PDMS under repeated mechanical loading, cyclic tensile and cyclic compression fatigue tests were performed. As shown in Figure S6A, during the cyclic tensile test, the sample was first stretched to a strain of approximately 100% and then unloaded to recover to its initial state. This process was repeated for 1000 cycles. Subsequently, tensile stress–strain tests were conducted on the cycled samples. As shown in Figure 4C,D, after 1000 cycles of tensile loading, the tensile strength of the DIW-printed porous PDMS decreased from 0.62 MPa to 0.52 MPa, with a tensile strength loss rate of approximately 16.1%. Its tensile strength was about 0.10 MPa lower than that of the uncycled sample. These results indicated that repeated tensile loading did not cause excessive damage to the porous network structure. The continuous PDMS framework (Figure 2C) constructed by DIW could withstand cyclic deformation to some extent while maintaining structural integrity.

In addition, cyclic compression tests were performed on DIW-printed porous PDMS. As shown in Figure S6B, the sample was compressed to a height of approximately 3 mm and then released, allowing it to undergo continuous compression–recovery cycles for 1000 cycles. After the cyclic test, compressive stress–strain tests were conducted on the sample. As shown in Figure 4E, stress softening (Mullins effect) occurred within the range of 20–40% after 1000 compression cycles. The Mullins effect is often observed in soft materials [62]. This phenomenon did not affect the structural integrity of the DIW-printed porous PDMS, but it significantly influenced its energy dissipation and stress relaxation. However, after 1000 compression cycles, the sample still maintained stable compressive resistance at 60% strain with a compressive strength of 23.3 kPa (Figure 4F), which was only approximately 0.4 kPa lower than that of the uncycled sample (23.7 kPa). This slight performance attenuation further confirmed the excellent fatigue resistance and mechanical stability of the porous structure.

The above results demonstrate that the porous PDMS constructed by DIW possesses good cyclic mechanical durability, which can be mainly attributed to its ordered porous network structure and flexible PDMS elastic skeleton. The periodic porous structure can effectively alleviate local stress concentration induced by external loading, while the elasticity of PDMS chain segments endows the material with excellent deformation recovery capability. Therefore, the DIW-printed porous PDMS not only exhibits stable mechanical properties but also shows great potential for long-term cyclic use.

### 3.5. DCM Absorption Capacity Analysis of Porous Silicone Rubber Composites

The absorption behavior of PDMS for DCM is mainly controlled by solvent diffusion and the swelling process of the polymer network. Due to the highly flexible Si–O–Si main chain structure of PDMS, large free volume, and good compatibility with low-polarity organic solvents, DCM molecules can diffuse into the cross-linked PDMS network. The absorbed solvent molecules occupy the free volume between polymer chains, increasing the distance between the chain segments, thereby inducing volume expansion of the PDMS network. This swelling process continues until the solvent mixing driving force reaches equilibrium with the elastic recovery force of the cross-linked network.

**Figure 5:**
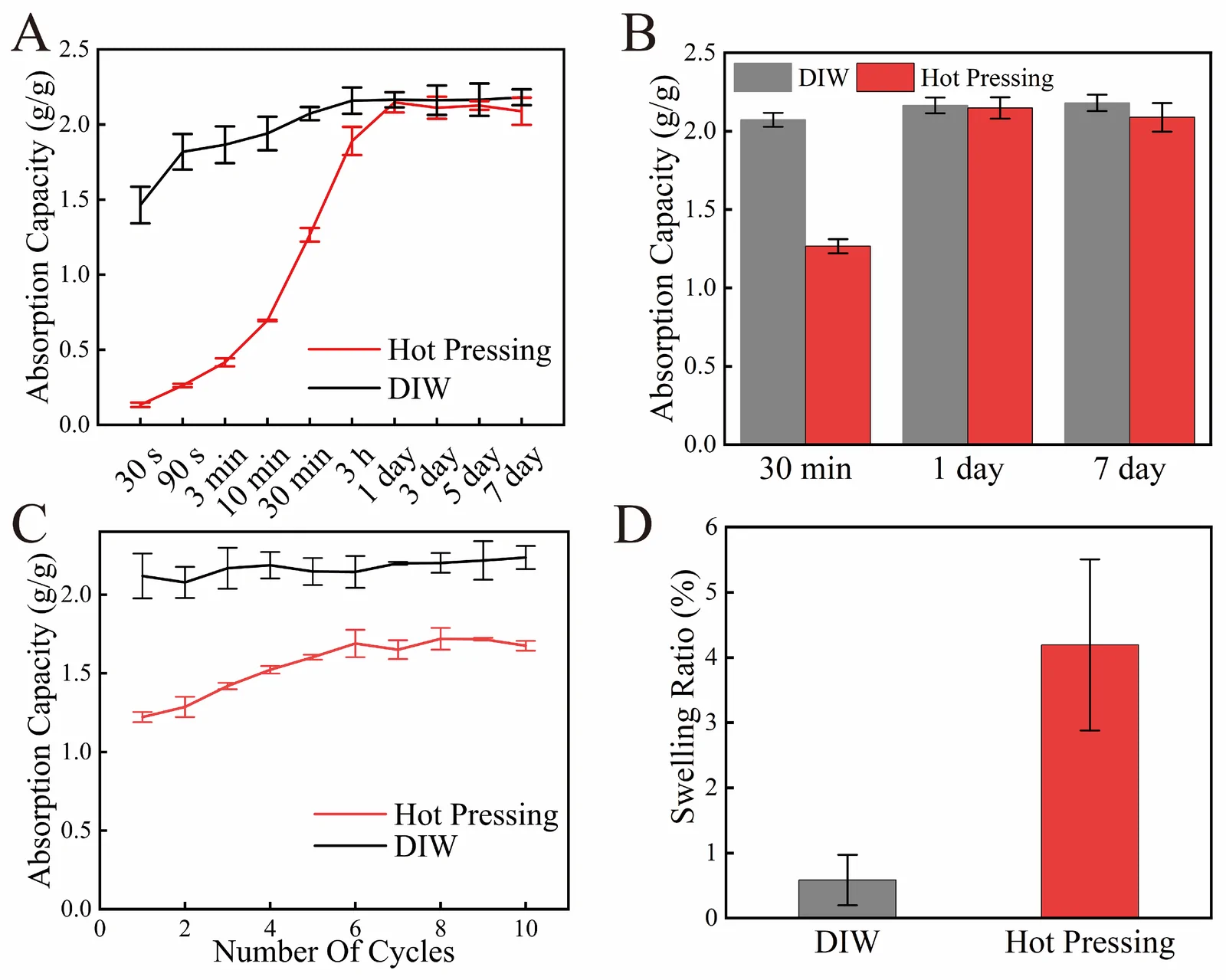
DCM absorption capacity of DIW-printed and hot-pressed silicone rubber at different absorption times (**A**). Comparison of the DCM absorption capacity of DIW-printed and hot-pressed silicone rubber after 30 min, 1 day, and 7 days of absorption (**B**). Cyclic DCM absorption capacity of DIW-printed and hot-pressed silicone rubber (**C**). Swelling ratio of DIW-printed and hot-pressed silicone rubber after immersion in water for 30 days (**D**).

To demonstrate the DCM absorption performance of DIW-printed PDMS, the time-dependent DCM absorption capacity, cyclic DCM absorption capacity, and water swelling behavior were investigated. Figure 5A compares the dynamic DCM absorption capacity of hot-pressed and DIW-printed PDMS. The first 30 min represented the rapid DCM absorption stage, during which a clear difference was observed between the two types of PDMS. Both samples showed an initial increase followed by gradual stabilization. At 30 min, the DCM absorption capacity of DIW-printed PDMS reached 2.07 ±0.04 g/g, whereas that of hot-pressed PDMS was only 1.27 ± 0.05 g/g. After 30 min, the DCM absorption capacity of DIW-printed PDMS tended to stabilize and reached a saturated state, whereas that of hot-pressed PDMS continued to increase slowly and did not approach saturation until after 1 day. Figure 5B compares the DCM absorption capacity of DIW-printed and hot-pressed PDMS at representative time points. It can be clearly observed that when DIW-printed PDMS reached saturation at 30 min, its DCM absorption capacity was significantly faster than that of hot-pressed PDMS. However, when hot-pressed PDMS reached saturation after 1 day, the difference between the two samples became relatively small. These results indicate that the DCM uptake rate of DIW-printed porous PDMS was much faster than that of conventional hot-pressed PDMS. This is because the porous structure printed by DIW can accommodate sufficient air, while significantly increasing the surface area of the material. Therefore, this microscopic-level pore structure acts as a rough structure, achieving superhydrophobicity/superoleophilicity. This wetting behavior helps to achieve rapid and selective absorption of DCM.

Figure 5C shows the cyclic DCM absorption capacity of PDMS prepared by DIW and hot pressing. With increasing cycle number, the DCM absorption capacity of hot-pressed PDMS increased until the sixth cycle. This phenomenon was mainly because this sample required 24 h to reach DCM absorption saturation. In addition, because of its dense structure, most of the absorbed DCM could not be squeezed out during each compression cycle. After the sixth cycle, the DCM absorption capacity tended to stabilize, indicating that the sample reached a relatively saturated state. In contrast, DIW-printed PDMS maintained a fast and stable DCM absorption capacity throughout the cycles. This indicates that the porous structure of the PDMS matrix was not obviously damaged during cyclic compression-assisted DCM absorption, which is consistent with the previous compression mechanical test results. Moreover, nearly all the absorbed DCM could be squeezed out during each compression process, allowing the sample to absorb fresh DCM again to the greatest extent and thereby enabling waste DCM recovery.

To further demonstrate that the material was not affected by water during DCM–water separation, water swelling experiments were performed on samples prepared by the two methods. As shown in Figure 5D, after immersion in ultrapure water for 30 days, the swelling ratios of both samples remained below 5%. Moreover, the water swelling ratio of DIW-printed porous PDMS was lower than that of conventional hot-pressed PDMS, which should be due to the higher water repellency of superhydrophobic DIW-printed porous PDMS compared with the hydrophobic hot-pressed PDMS, further confirming that DIW-printed porous PDMS is more suitable for waste DCM recovery.

Figure S7A,B show optical images of DCM absorption tests using 10 × 10 mm PDMS samples prepared by DIW and hot pressing, respectively, in a mixture of Sudan IV-stained DCM and water. The DIW-printed PDMS rapidly absorbed DCM immediately after contact and turned completely red (Figure S7A and Video S1, video attached in the Supporting Information). In contrast, the hot-pressed PDMS still failed to fully absorb the DCM after more than 1 min of contact with DCM, and the absorbed DCM was unevenly distributed (Figure S7B and Video S2, video attached in the Supporting Information). This further demonstrates that the DCM uptake rate of DIW-printed PDMS was much faster than that of conventional hot-pressed PDMS.

The difference in DCM uptake rate between the two types of PDMS essentially originates from the different pore structures generated by the fabrication methods. DIW 3D printing enables precise regulation of the pore structure and allows the construction of an ordered porous architecture with high connectivity. DCM can rapidly diffuse through the three-dimensionally interconnected channels, thereby greatly shortening the time required to reach absorption equilibrium and significantly improving the DCM uptake rate. In contrast, owing to the limitations of the hot-pressing process, the hot-pressed PDMS has almost no interconnected pores, resulting in high resistance to DCM penetration. Consequently, it exhibits a slower DCM uptake rate and requires a longer time to reach DCM absorption saturation.

**Figure 6:**
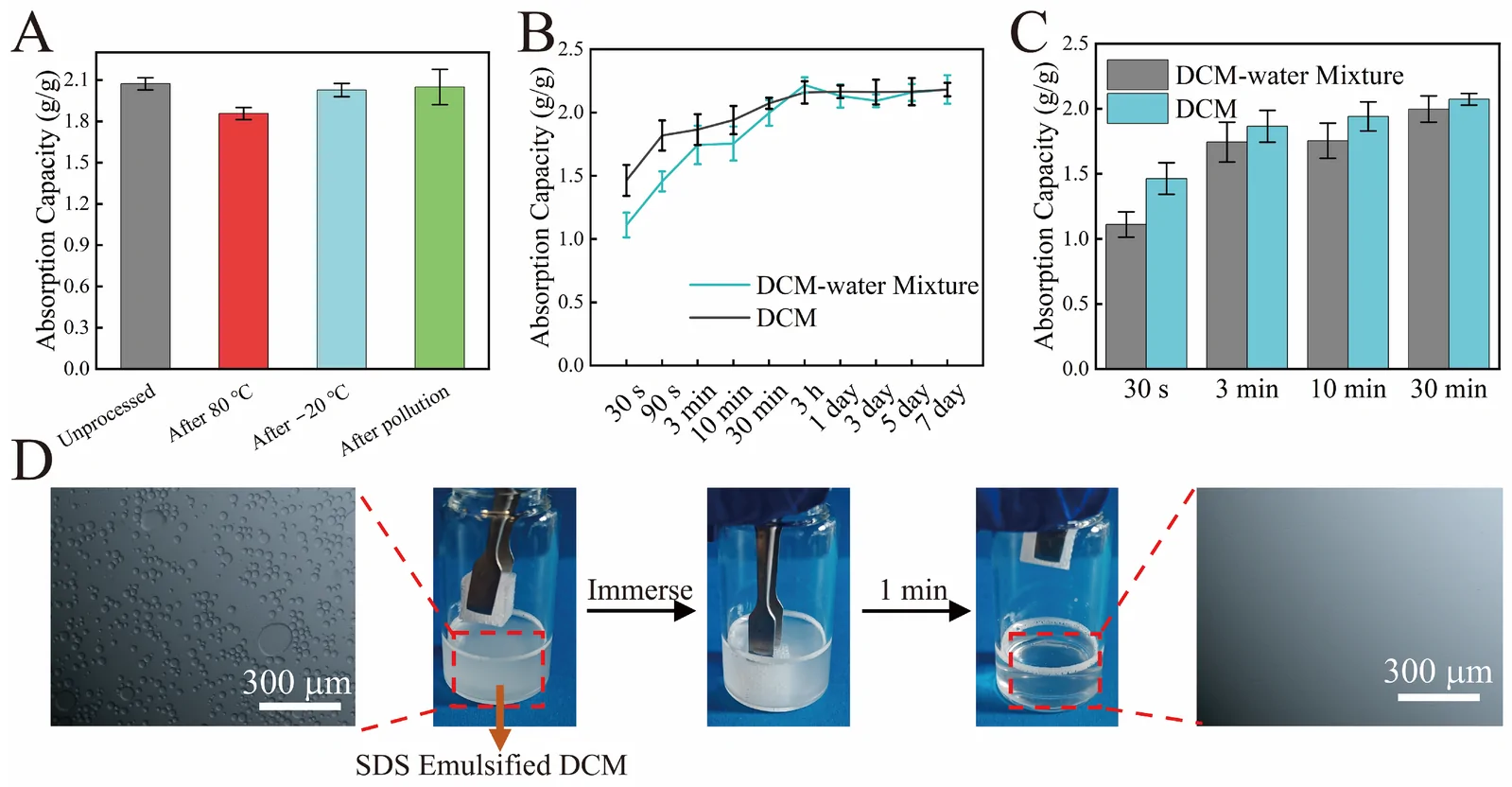
Dependence of absorption capacity on immersion medium, temperature (**A**), immersion time (**B**), and composition (**C**). Microscopic image of SDS-emulsified DCM in water (**D**).

In practical DCM–water separation applications, absorbent materials are inevitably affected by complex environmental factors, such as pollutants and temperature variations. Therefore, to evaluate the stability of DIW-printed porous PDMS under different environmental conditions, the samples were treated in a pollutant environment at a high temperature of 80 °C and at a low temperature of −20 °C for 12 h, respectively, followed by testing their DCM absorption performance. As shown in Figure S8, the DCM absorption regeneration performance after pore blockage was specifically designed and characterized in a mixed pollutant system with various typical pollutants, simulating pore blockage. The DIW-printed porous PDMS samples were buried in the mixed pollutant system for 12 h to simulate a complex pollutant environment in practical applications. Subsequently, the samples treated under different environmental conditions were immersed in DCM for 30 min, and their DCM absorption capacity was measured. As shown in Figure 6A, compared with the untreated sample, DIW-printed porous PDMS treated under different environmental conditions still maintained fast DCM absorption capacity. Among them, the sample treated at 80 °C showed the most obvious decrease in DCM absorption capacity, but it still maintained a relatively high level, with only a decrease of approximately 0.22 g/g. In contrast, the DCM absorption capacity of the samples treated in the pollutant environment and at −20 °C decreased by only 0.02 g/g and 0.04 g/g, respectively. These results indicate that DIW-printed porous PDMS possesses excellent environmental tolerance. Its porous structure and PDMS elastic network can remain stable under different temperature and pollutant conditions, thereby ensuring long-term DCM absorption performance. This good environmental adaptability provides reliable support for its application in complex practical DCM–water separation environments.

To further investigate the separation capability of DIW-printed porous PDMS in a practical DCM–water system, the material was placed in a DCM–water mixture for dynamic DCM absorption tests. As shown in Figure 6B, compared with the pure DCM system, DIW-printed porous PDMS exhibited a slightly lower initial DCM uptake rate in the DCM–water mixture. However, with increasing absorption time, the DCM absorption capacities in the two systems gradually stabilized and became comparable. Specifically, within 30 s, the DCM absorption capacity reached 1.46 g/g in the pure DCM system, whereas it was 1.11 g/g in the DCM/water mixture, showing a difference of 0.35 g/g. As the absorption process continued, the DCM absorption capacities in the pure DCM and DCM–water systems reached 2.07 and 2.00 g/g after 30 min, respectively, with only a small difference of 0.07 g/g (Figure 6C). The lower initial DCM uptake rate in the DCM–water mixture may be attributed to the partial hindrance of DCM mass transfer by the aqueous phase. At the DCM–water interface, DCM must first undergo interfacial migration before entering the internal pores of PDMS, while the presence of water reduces the probability of direct contact between DCM molecules and the absorbent. However, with prolonged absorption time, DCM gradually entered the internal pores and was efficiently absorbed because of the high affinity of PDMS for organic solvents and the continuous capillary channels provided by the porous network. Therefore, the final absorption capacity was close to that obtained in the pure DCM system, demonstrating the excellent selective DCM absorption capability of DIW-printed porous PDMS.

In addition, to further verify the ability of DIW-printed porous PDMS to remove trace DCM phases, SDS was used as a surfactant to prepare a stable DCM-in-water emulsion. The emulsified mixture appeared visibly milky white. Optical microscopy revealed the presence of numerous small DCM droplets stabilized by the surfactant in the system (Figure 6D). Subsequently, DIW-printed porous PDMS was immersed in the emulsion system. After 1 min of treatment, the emulsion rapidly changed from a turbid state to a transparent and clear state, and no obvious residual DCM droplets were observed by optical microscopy, indicating nearly complete DCM-water separation (Video S3, video attached in the Supporting Information). These results indicate that the porous PDMS could effectively remove trace dispersed DCM phases.

## 4. Conclusions

In this study, functional porous silicone rubber integrating ASW, superelasticity, and DCM absorption performance was successfully fabricated by DIW 3D printing using PDMS as the matrix and by precisely regulating printing geometric parameters, including printing speed, pneumatic pressure, and nozzle angle. The obtained material exhibited pronounced ASW and showed superhydrophobicity in the X direction. The DIW-printed porous PDMS maintained structural integrity without a distinct failure point under large strain and rapidly recovered its original shape upon unloading. After cyclic tensile and compressive loading, it showed only a modest reduction in tensile strength and negligible compressive degradation, indicating durable cyclic mechanical stability. In DCM absorption, the DIW sample exhibited faster early-stage uptake than conventional hot-pressed PDMS while achieving comparable equilibrium absorption capacity. This study integrates ASW, superelasticity, and recyclable DCM absorption into a single 3D-printed porous silicone rubber system and provides new strategies for the design and construction of materials for waste DCM recovery, DCM-water separation, waterproof and breathable membranes, and flexible biomedical materials. These findings also provide theoretical guidance and an experimental basis for the development of next-generation multifunctional silicone rubber materials. However, its DCM absorption capacity and applicability in biomedical applications still need to be further improved. In future research, strategies such as salt leaching can be considered to increase the porosity, thereby further enhancing its DCM absorption capacity. For biomedical applications, the material surface can be modified by adding hydrophilic coatings, such as hydrogels, to meet the hydrophilic requirements of biomedical applications.

## Data Availability

Data will be made available on request.

## Supplementary Materials

The following supporting information can be downloaded at https://www.mdpi.com/article/10.3390/polym18212714/s1. Table S1: Performance comparison between this study and other typical PDMS-based porous materials for DCM–water separation. Figure S1: Construction and applications of multifunctional silicone rubber by DIW 3D printing (A). The process of preparing PDMS by DIW and hot pressing (B). Figure S2: Porous silicone rubber structures printed under different pneumatic pressures: 4.0 bar (A), 3.7 bar (B), 3.4 bar (C), 3.1 bar (D), 2.8 bar (E), and 2.5 bar (F). Structures printed using the slant nozzle (G) and the vertical nozzle (H). Figure S3: Time-dependent WCA images of samples before swelling (A) and after swelling (B) for X direction, Y direc-tion, and silicone rubber prepared by hot pressing. Figure S4: Rolling angle measured in the X direction (A) and Y direction (B). Optical images showing the hydrophobicity (C) and oleophilicity (D) of DIW-printed porous PDMS. Figure S5: Photographs showing the compressed state of the sample during the compression test (A). Photographs of DIW-printed porous PDMS before (left) and after (right) the compression test (B). Photographs showing the stretched state of the sample during the tensile test (C). Photograph showing the tensile resistance of DIW-printed porous PDMS under a 150 g load (D). Photograph showing the bending test of DIW-printed porous PDMS (E) and hot-pressed PDMS (F) under a 500 g load. Figure S6: Schematic illustration of cyclic tensile loading (A) and cyclic compressive loading (B) on DIW-printed porous PDMS. Figure S7: Optical images showing the DCM absorption process of DIW-printed porous silicone rubber (A) and hot-pressed silicone rubber (B) in a mixture of Sudan IV-stained DCM and deionized water. Figure S8: Process of immersing DIW-printed porous PDMS in a mixed pollutant system.
