# Supplementary material for: Direct Ink Writing 3D Printing of Anisotropic Superhydrophobic/Superelastic Porous Silicone Rubber for Recyclable Dichloromethane Absorption
Source: Polymers (Basel). 2026 Nov 2;18(21):2714. doi: 10.3390/polym18212714 (PMC13766087; doi:10.3390/polym18212714)
Supplement: Supplementary file 1 [file polymers-18-02714-s001.zip › polymers-4587484-supplementary.pdf]

polymers

Direct Ink Writing 3D Printing of Anisotropic Superhydrophobic/Superelastic Porous Silicone Rubber for Recyclable Dichloromethane Absorption

Longtao Ge [a, b, #], Zhoukun He [a, b, #], Lin Deng [a, b, #], Zhijun Wang [b], Qiang Guo [c, d], David Hui [e], Wei Feng [a], Qingyuan Wang [a, b, f, *], Xiaorong Lan [c, d, g, h, *] and Jiayan Mao [i,*]

a. School of Mechanical Engineering, Chengdu University, Chengdu, 610106, China.
b. Institute for Advanced Study, Research Center of Composites & Surface and Interface Engineering, Chengdu University, Chengdu, 610106, China.
c. Luzhou Key Laboratory of Oral & Maxillofacial Reconstruction and Regeneration, The Affiliated Stomatological Hospital, Southwest Medical University, Luzhou, 646000, China.
d. Institute of Stomatology, Southwest Medical University, Luzhou, 646000, China.
e. Department of Mechanical Engineering, LSU New Orleans, Louisiana, 70148, USA.
f. Failure Mechanics and Engineering Disaster Prevention Key Laboratory of Sichuan Province, Sichuan University, Chengdu, 610065, China.
g. Basic Medicine Research Innovation Center for Cardiometabolic Diseases, Ministry of Education, Southwest Medical University, Luzhou, 646000, China.
h. Metabolic Vascular Diseases Key Laboratory of Sichuan Province, Southwest Medical University, Luzhou, 646000, China.
i. Department of Nephrology and Institute of Nephrology, Sichuan Provincial People's Hospital, School of Medicine, University of Electronic Science and Technology of China, Sichuan Clinical Research Centre for Kidney Diseases, Chengdu, 610072, China.

* Correspondence: Corresponding authors: Qingyuan Wang: wangqy@scu.edu.cn, +86 13980955902; Xiaorong Lan: xiaoronglan@swmu.edu.cn, +86 18384297885; Jiayan Mao: maojiayan123@126.com, +86 13980880849
#. These authors contributed equally to this work

Table S1. Performance comparison between this study and other typical PDMS-based porous materials for DCM–water separation

Fabrication method	Anisotropic Surface Wettability (ASW)	Precision of structural/pore regulation	Mechanical fatigue	Uptake or separation rate	Reference
DIW-printed PDMS	Exhibited ASW behavior, with a WCA difference of 25.9°	Both the overall geometry and pore structure could be digitally and precisely regulated, enabling deterministic structural construction	Exhibited superelasticity, with no obvious strength attenuation or shape change after 1000 loading cycles	2.07 g/g uptake rate for DCM	This study
Porous PDMS prepared by water-in-silicone DCM emulsion/soft-template method	Directional wetting design was not discussed	The structure and pore size could not be precisely regulated	Exhibited good compression recovery, but structural collapse was observed after 1000 compression cycles at 60% strain	10 g/g uptake rate for DCM	[51]
Magnetic particle/PDMS composite prepared by foaming and templating	Exhibited certain ASW, with a WCA difference of 10.4°	The pore structure was mainly determined by the template, and the pore size and architecture could not be precisely regulated	After 30 cyclic compression tests, the residual height of the aerogel was 87%, indicating certain deformation	49 g/g uptake rate for DCM	[52]
Graphene-reinforced porous PDMS constructed by a template method	Directional wetting design was not discussed	The pore structure depended on the template, making precise design of pore-channel arrangement difficult	No obvious plastic deformation or stress loss was observed after 100 compression cycles at 80% strain	14 g/g uptake rate for DCM	[53]
Commercial sponge modified by PDMS coating combined with SiO_2 and metal–organic framework (MOF) particles	Directional wetting design was not discussed	The pore structure was mainly inherited from the sponge substrate, with regulation primarily achieved by surface functionalization	Exhibited certain mechanical stability, but long-term fatigue performance was not systematically investigated	21 g/g uptake rate for DCM	[54]

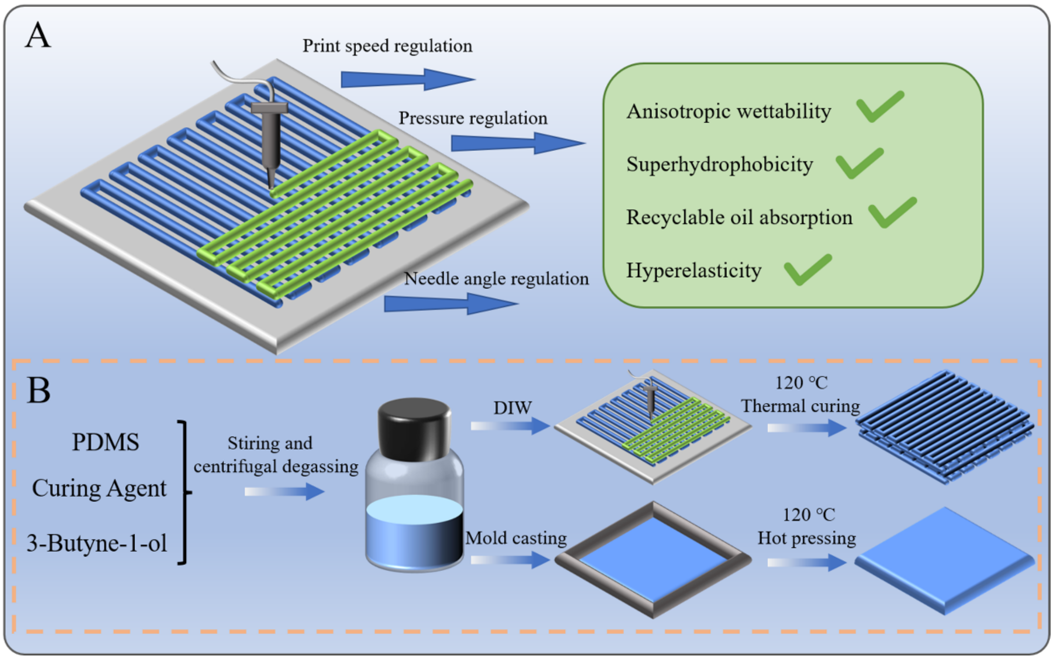

Figure S1. Construction and applications of multifunctional silicone rubber by DIW 3D printing (A). The process of preparing PDMS by DIW and hot pressing (B).

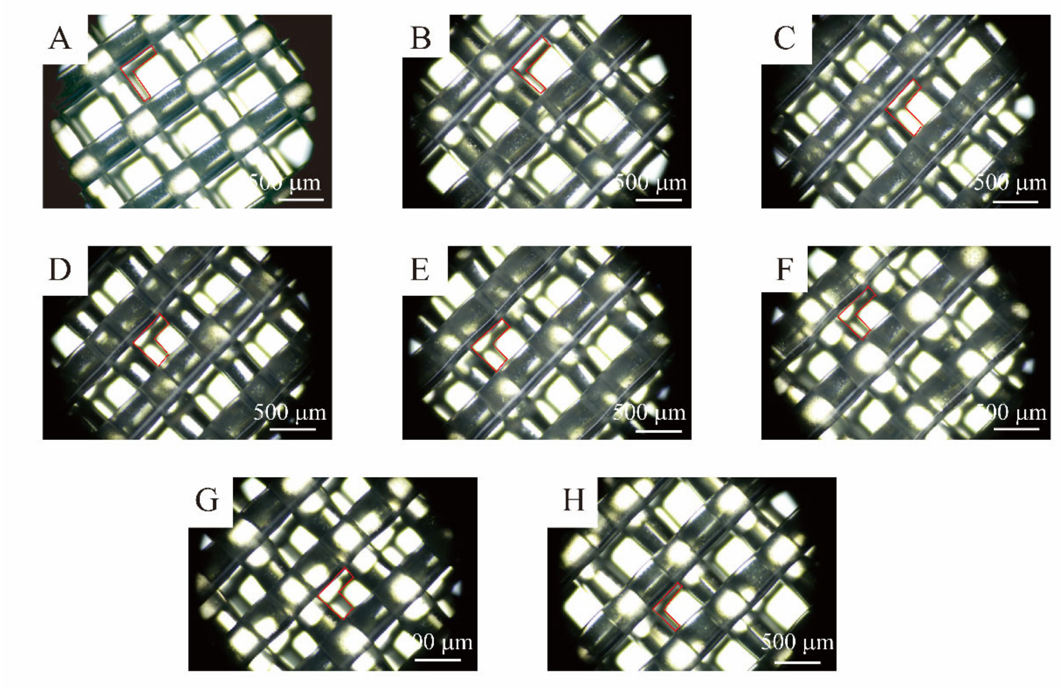

Figure S2. Porous silicone rubber structures printed under different pneumatic pressures: 4.0 bar (A), 3.7 bar (B), 3.4 bar (C), 3.1 bar (D), 2.8 bar (E), and 2.5 bar (F). Structures printed using the slant nozzle (G) and the vertical nozzle (H).

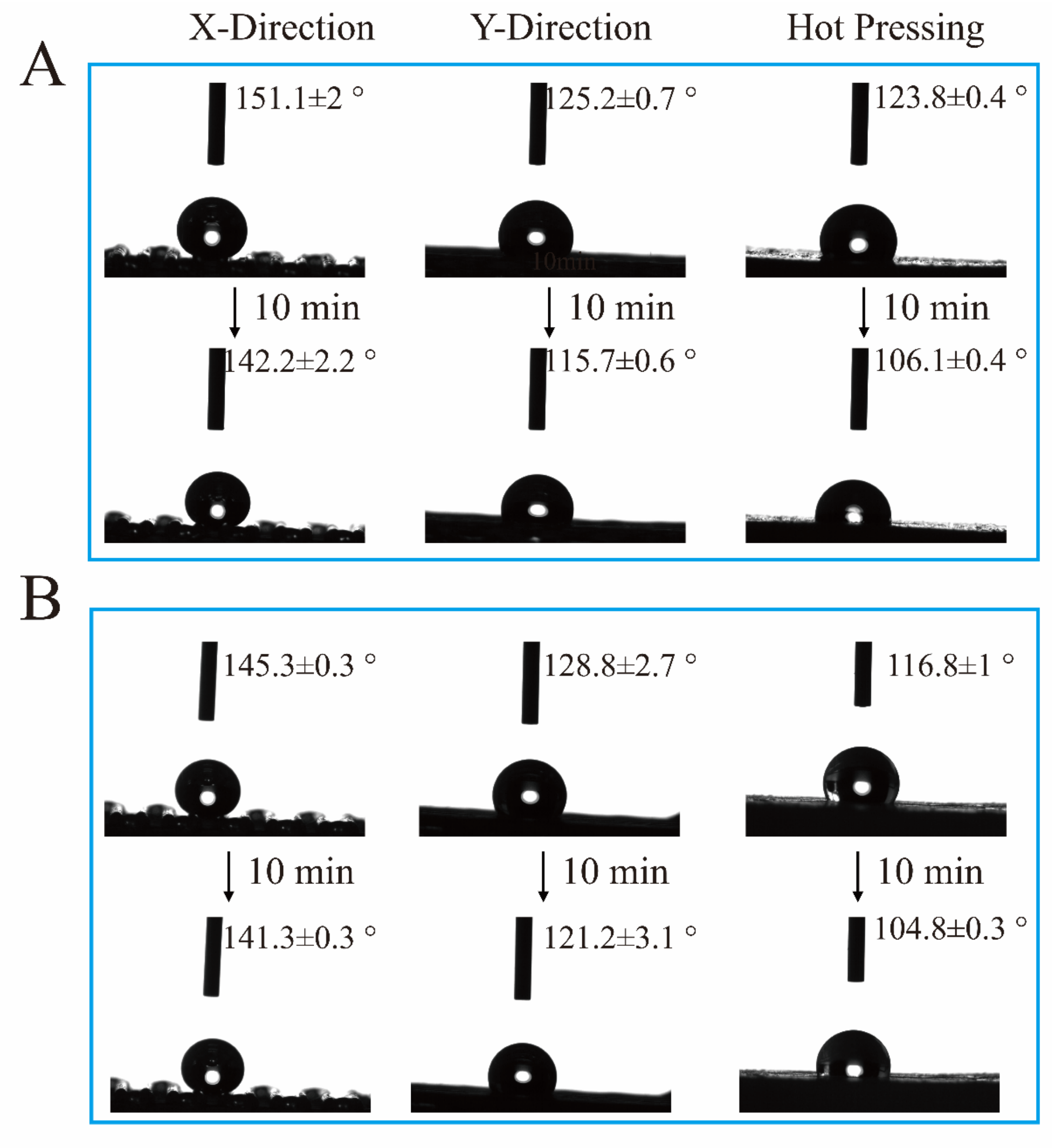

Figure S3. Time-dependent WCA images of samples before swelling (A) and after swelling (B) for X direction, Y direction, and silicone rubber prepared by hot pressing.

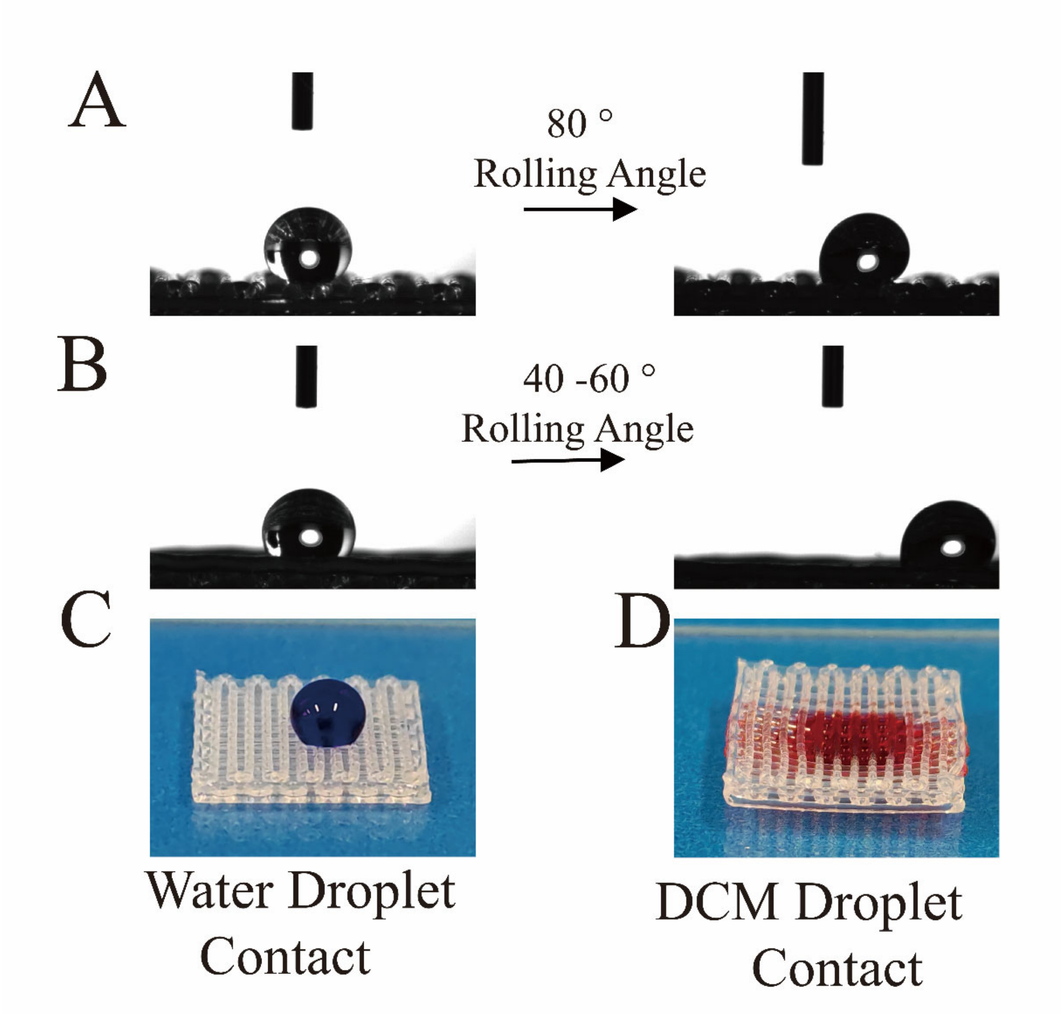

Figure S4. Rolling angle measured in the X direction (A) and Y direction (B). Optical images showing the hydrophobicity (C) and oleophilicity (D) of DIW-printed porous PDMS.

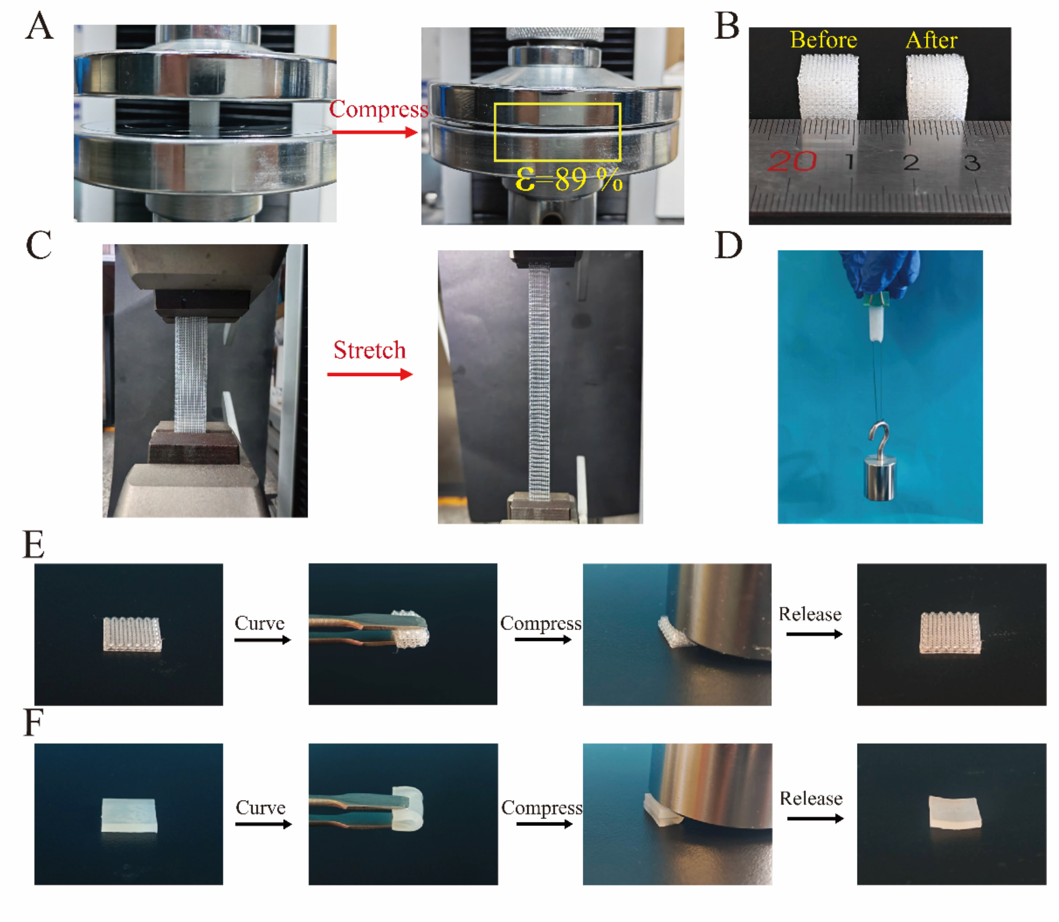

Figure S5. Photographs showing the compressed state of the sample during the compression test (A). Photographs of DIW-printed porous PDMS before (left) and after (right) the compression test (B). Photographs showing the stretched state of the sample during the tensile test (C). Photograph showing the tensile resistance of DIW-printed porous PDMS under a 150 g load (D). Photograph showing the bending test of DIW-printed porous PDMS (E) and hot-pressed PDMS (F) under a 500 g load.

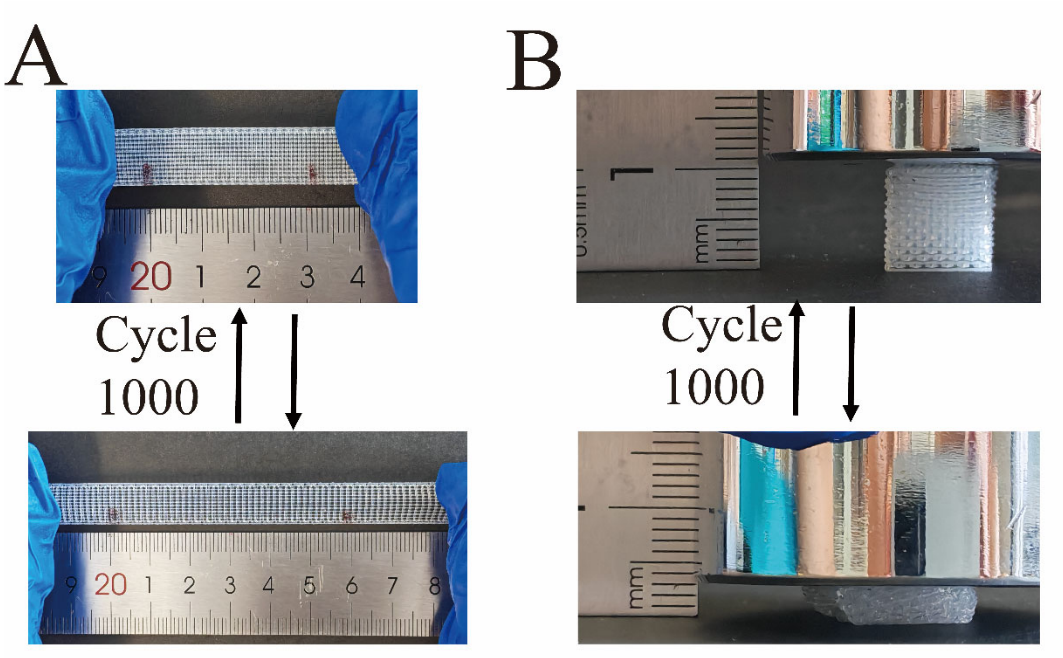

Figure S6. Schematic illustration of cyclic tensile loading (A) and cyclic compressive loading (B) on DIW-printed porous PDMS.

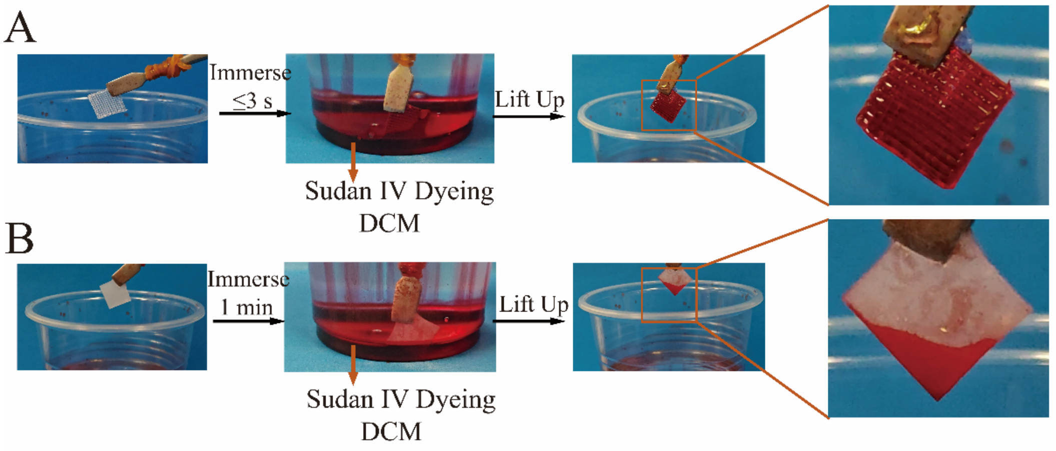

Figure S7. Optical images showing the DCM absorption process of DIW-printed porous silicone rubber (A) and hot-pressed silicone rubber (B) in a mixture of Sudan IV-stained DCM and deionized water.

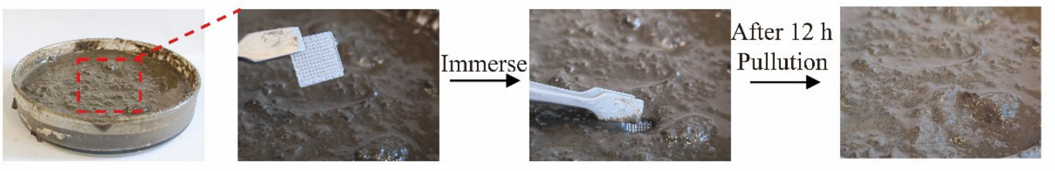

Figure S8. Process of immersing DIW-printed porous PDMS in a mixed pollutant system.
